# Treg-targeted efficient-inducible platform for collagen-induced arthritis treatment

**DOI:** 10.1016/j.mtbio.2023.100557

**Published:** 2023-01-20

**Authors:** Lin Wang, Yi Wang, Chang Liu, Jiachen He, Xu He, Xiongjinfu Zhang, Can Zhu, Jie Sun, Qin Wang, Hao Chen, Qin Shi

**Affiliations:** a Department of Orthopedics, The First Affiliated Hospital of Soochow University, Orthopedic Institute of Soochow University, Suzhou Medical College of Soochow University, 899 Pinghai Road, Suzhou, Jiangsu, 215031, PR China; b Department of Immunology, School of Biology & Basic Medical Sciences, Suzhou Medical College of Soochow University, 199 Renai Road, Suzhou, Jiangsu, 215021, PR China; c Department of Orthopedics, Affiliated Hospital of Yangzhou University, Yangzhou University, No. 368, Hanjiang Middle Rd, Yangzhou, Jiangsu, 225000, PR China; d Department of Orthopedics, Wuxi Ninth People's Hospital Affiliated to Soochow University, Wuxi, Jiangsu, 214026, PR China

**Keywords:** Rheumatoid arthritis, Treg cells, Nanoparticle drug delivery system, Treg/Th17

## Abstract

Regulatory T cells (Tregs) display great promise in rheumatoid arthritis (RA) therapy. However, their low number and differentiation rate limit their further application in the clinics. In the present study, we first optimized a combination of IL-2, TGF-β and cyclin dependent kinase inhibitor AS2863619 (IL-2/TGF-β/AS), which could induce Tregs with high efficiency *in vitro*. After the induced Tregs (iTregs) were confirmed to suppress lymphocyte proliferation and pro-inflammatory T help cells (Th1 and Th17) activation, a chitosan-stabilized nanoparticle drug delivery system (NDDS) was developed according to the optimized formula of IL-2/TGF-β/AS. *In vivo* study, the NDDS was injected into the knees of mice with collagen-induced arthritis (CIA). As a result, the NDDS remarkably reduced the pathological score of the CIA, alleviated the inflammatory cell infiltration and synovial hyperplasia, and minimized cartilage tissue damage in the knee joint of the CIA mice. Mechanically, the NDDS administration promoted Treg differentiation and decreased Th17 production, consequently reversing the ratio of Treg/Th17, and reducing the secretion of TNF-α in the sera, which facilitated to relieve the severity and progression of arthritis. In sum, NDDS capable of efficiently inducing Tregs were constructed successfully and provided a potential platform for treating RA by restoring the equilibrium of Treg/Th17 destroyed in RA.

## Graphical abstract

Treg-targeted nanoparticle drug delivery system rescues the imbalance of Th1/Th2 and Treg/Th17 impaired in rheumatoid arthritis.

**Figure:**
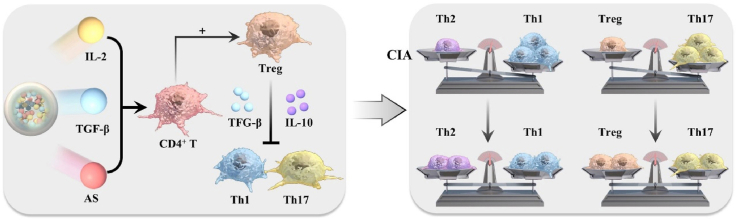

## 1 Introduction

Rheumatoid arthritis (RA) is a persistent, systemic inflammatory disease, characterized by inflammation of the synovial membrane of the joint cavity, the lining layer thickening, and significant lymphocyte infiltration, eventually resulting in joint deformity and loss of function [1,2]. Though there is currently no consensus regarding the cause of RA, it is acceptable that a significant proportion of CD4^+^T cells enter the synovial tissue, initiating the development of RA [3,4]. Primed by autoantigen, CD4^+^T cells are overactivated and most of them differentiated into inflammatory cells such as T helper 1 ​cell (Th1) and T helper 17 ​cell (Th17), and the proportion of differentiation into Treg cells was relatively reduced. Thus, redundant Th1 and Th17 ​cells impair the equilibrium of Th1/T helper 2 ​cell (Th2) and Tregs/Th17 ​cells, respectively, which is usually in dynamic balance under the physiological conditions. To this end, tumor necrosis factor-α (TNF-α), interferon-γ (IFN-γ), and interleukin-17A (IL-17A) are secreted in tremendous amounts, which puts the joint continuously in a state of chronic inflammation [5,6], and causes synovial hyperplasia, pannus development, osteoclast activation, and ultimately the cartilage damage and subchondral bone loss [7,8]. In the chronic course of RA, inflammatory cytokines increase while anti-inflammatory cytokines are secreted inefficiently. Therefore, Th1/Th2 and Treg/Th17 ​cell imbalance plays an important role in the occurrence and development of RA. Nonsteroidal anti-inflammatory drugs [5], glucocorticoids [6], and antirheumatic drugs [7] are commonly applied to treat RA in order to reduce inflammation and relieve pain. However, usage of these medications has the potential to harm the livers and kidneys as well as the gastrointestinal tract [8]. Due to the advantages of the high selectivity of the pharmacological action and low toxic side effects, biological agents have attracted broad attention as a promising therapy in RA [9].

Tregs are characterized by strong immunosuppressive properties to maintain immunological homeostasis by controlling peripheral immune tolerance. They are identified by the expression of Foxp3, CD4, and CD25. Through intercellular interaction, Tregs consistently block the cytotoxic effects of CD8^+^T cells and NK cells and the activation and proliferation of autoreactive T cells by producing inhibitory cytokines, such as transforming growth factor-β (TGF-β) and interleukin-10 (IL-10) [10,11]. It is noted that, in contrast to the nature Tregs (nTregs) derived from the thymus, the inducible Tregs (iTregs) are more able to prevent the proliferation of synovial fibroblasts and minimize the secretion of inflammatory cytokines in the RA mice [12]. Accumulative evidence further supports that the number of Tregs is related to the clinical parameters in RA patients [13]**.** Consequently, Tregs have been a target of cell-based therapies with new biological medicines to treat RA [12].

To our knowledge, interleukin-2 (IL-2) and TGF-β are the significant agents employed to generate iTregs. However, the activity of iTregs following expansion is obviously impacted by the prolonged induction duration and low induction efficiency [14]. AS2863619 (AS) is a small molecule inhibitor of cyclin-dependent kinase (CDK) 8 and 19 with a relative molecular weight of 405.24. It inhibits serine phosphorylation in the PSP motifs of signal transducer and activator of transcription 5 (STAT5) and somehow enhances phosphorylation of tyrosine residues in the C-terminal domain, leading to enhanced activation of STAT5 and thus activation of various STAT5-binding genes, including Treg signature genes such as Foxp3 and CD25 [15]. Thus, finding a new formula with AS, not only increases the induction efficiency of iTregs, but also improves the inhibitory function, which are therefore of critical significance in RA therapy.

With the development of biomaterials, drug delivery strategies for treating disease have been substantially well-designed and applied. Reasonable drug “packing” can not only enhance its pharmacokinetics as well as its solubility and bioavailability, but also reduce side effects by controlling their release speed [16,17]. A nanoparticle delivery system with a particle size of 1–1000 ​nm, such as nanoparticles, nanocapsules, and nanoliposomes, is well-established and proved by Food and Drug Administration (FDA) [18]. Drugs are loaded into the nanoparticles by means of either physical or chemical techniques. Bovine serum albumin (BSA) has been utilized extensively as a drug delivery vehicle due to its excellent biocompatibility and biodegradability [19,20]. For instance, Abraxane, an FDA-approved paclitaxel albumin-binding nanoparticle, is delivered to treat metastatic breast cancer. Carboplatin, used in metastatic non-small cell lung cancer, is a sufficient example of a safe and efficient nanoparticle immunotherapy strategy [21]. By joining BSA to the E7 antigen, Zhang L et al. successfully created a new human papillomavirus nanovaccine that induced dendritic cell maturation and boosted T-cell effectors, increasing anti-tumor immunity [22].

**Scheme 1:**
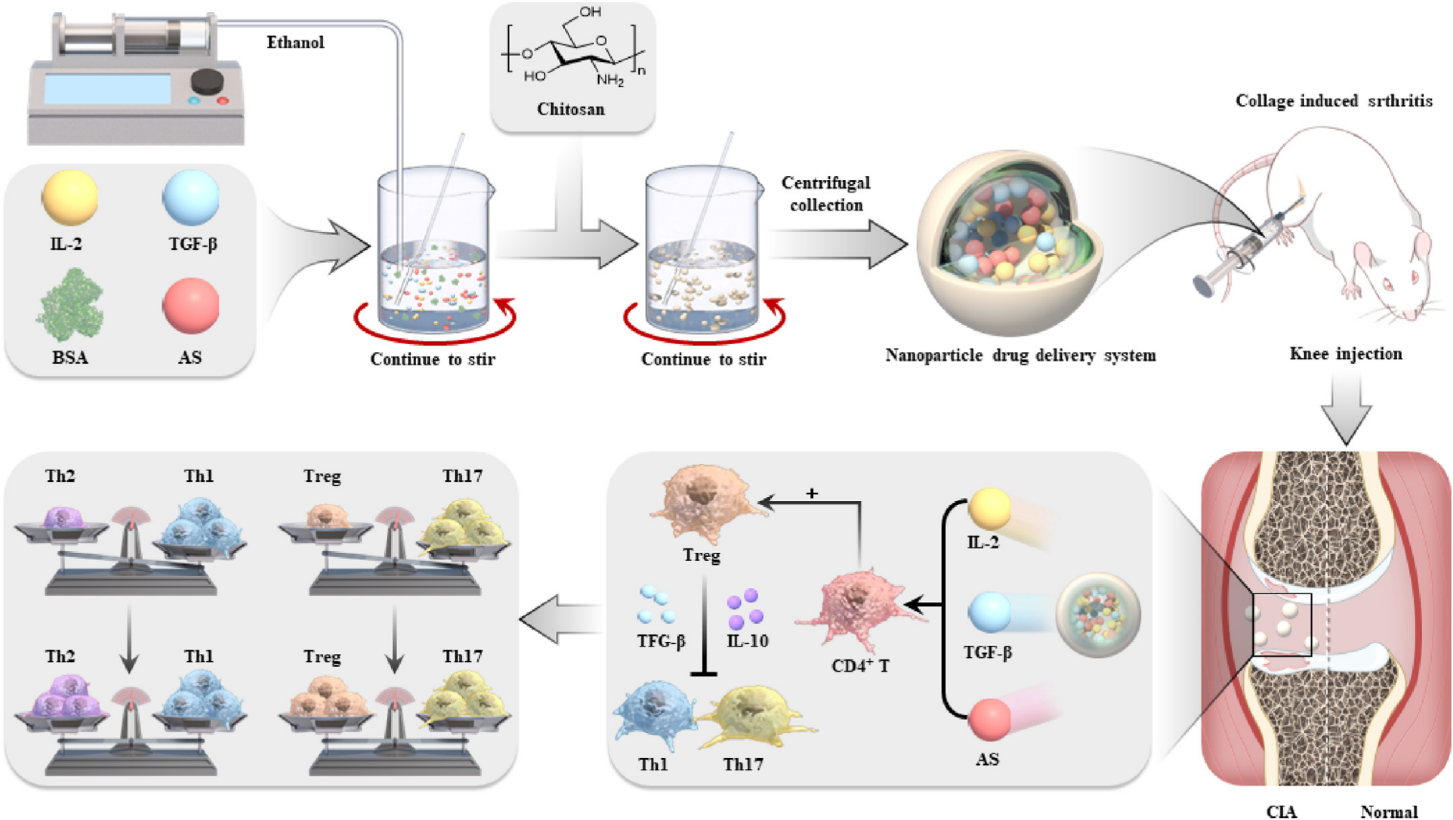
Chitosan-stabilized BSA nanoparticle drug delivery system (NDDS) was developed according to an optimized formula of IL-2/TGF-β/AS, and rescued the imbalance of Th1/Th2 and Treg/Th17 impaired in the rheumatoid arthritis.

In the present study, we first set up an optimized combinations with IL-2, TGF-β and AS2863619 (IL-2/TGF-β/AS) to induce mouse Tregs at high efficiency. Then, chitosan-stabilized BSA nanoscaled particles loaded with the formula of IL-2/TGF-β/AS were applied to fabricate a nanoparticle drug delivery system (NDDS). As last, the therapeutic effect of the nanoparticles was explored by injected to the knees of the mice with collagen-induced arthritis (CIA). Upon the administration of the NDDS, the severity of arthritis was mitigated, demonstrated by the reduced the pathological score, the decreased inflammatory cell infiltration and synovial hyperplasia (Scheme 1). Mechanically, the NDDS improved Treg differentiation efficiently and constantly, restored the balance of Treg/Th17 which was impaired in RA, and decreased TNF-α secretion in the sera. Therefore, NDDS is expected to be a promising and convenient platform for treating RA in the near future.

## 2 Methods

### 2.1 Mice and reagents

DBA/1 mice and BALB/c mice were obtained from SLAC LABORATORY ANIMAL (Shanghai, China). Six-week-old male mice were used for all experiments. All animal studies were approved by the Subcommittee on Research and Animal Care (SRAC) of Soochow University and were performed by following the guidelines of the Experimental Animal Center of Soochow University. The source of the reagents and antibodies used in this study were listed in the Supplementary Table 1.

### 2.2 Induction of iTreg cells *in vitro*

CD4^+^T cells were sorted from the spleen of the DBA/1 mice with mouse CD4 MicroBeads (Miltenyi Biotec, Germany) and cultured in the complete RPMI 1640 medium (10% fetal calf serum). Sorted CD4^+^T cells were stimulated with Ultra-LEAF™ purified anti-mouse CD3*ε* and CD28 antibodies (5 ​μg/mL, respectively). Tregs were induced by adding corresponding reagents, IL-2, TGF-β and/or different concentrations of AS for 48 ​h.

### 2.3 Flow cytometry assay (FCA)

Cells were collected and stained with anti-CD4, CD25 and Foxp3 antibodies following the instruction of the manufactory. For intracellular IFN-γ, IL-2 and IL-17A staining, the cells were stimulated in Cell Activation Cocktail at 37 ​°C for 4 ​h before being stained with the antibodies against to the markers. Stained cells were read on flow cytometry (Thermo Fisher, USA), and data were analyzed with FlowJo V10 software (BD, USA).

### 2.4 Quantitative real-time PCR (qRT-PCR)

Total RNA was extracted by Trizol and quantified with NanoDrop (Thermo Fisher, USA). Then, RNA was reverse-transcripted into cDNA and subjected to qRT-PCR with SYBR Quantitative Premix Kit (Bio-Rad, USA). The relative RNA expression (fold changes) of target genes were normalized by house-keeping gene GAPDH and calculated with the 2^−ΔΔCt^ method. The sequence of the primers was listed in the Supplementary Table 2.

### 2.5 Proliferation of lymphocytes

CD4^+^T cells were purified from the spleens of DBA/1 mice, and Tregs were induced with IL-2 (10 ​ng/mL), TGF-β (10 ​ng/mL) and AS (0–1000 ​nM). Ficoll-isolated lymphocytes (LYM) were extracted from the splenocytes of BALB/c mice and labeled with CFSE (5 ​μM). Then, iTreg and LYM were co-cultured at the ratio of 0:1, 1:8, 1:4, 1:2, and 1:1, respectively. After 72 ​h of culture, the proliferation of lymphocytes and activated T cells were detected by FCA.

### 2.6 Lymphocytes co-cultured with iTregs and the supernatant of iTregs

Tregs were induced for 48 h and harvested. Some of the iTreg cells were treated with mitomycin C (20 ​μg/mL). The others were further cultured in the culture medium deprived of cytokines, and the supernatant of iTregs was collected after 24 h. Meanwhile, lymphocytes prepared from the spleen of DBA/1 mice were co-cultured with mitomycin C-pretreated iTreg or the supernatant of iTregs with IL-2 (20 ​ng/mL), IL-6 (20 ​ng/mL), IL-12 (20 ​ng/mL) and TGF-β (5 ​ng/mL). Cultured for 48 ​h, the cells were collected, and IFN-γ and IL-17A in CD4^+^ T cells were detected by FCA.

### 2.7 Fabrication of nanoparticle drug delivery system

The nanoparticle drug delivery system was fabricated according to the previous work [23]. In brief, 50 ​mg of BSA in 5 ​mL of deionized water was stirred constantly. 20 ​mL of absolute ethanol was pumped evenly into the stirring BSA solution at a rate of 2 ​mL/min with a syringe pump. After stirring overnight, the BSA solution was pumped with 20 ​mL chitosan acetic acid solution (20 ​mg chitosan dissolved in 20 ​mL 1% acetic acid solution) at a speed of 2 ​mL/min. Afterward, 5 ​mL of absolute ethanol was pumped into the mixed solution at a speed of 0.5 ​mL/min. After stirring for 8 ​h, chitosan-stabilized BSA nanoparticles were obtained. When prepared the nanoparticle drug delivery system of IL-2/TGF-β/AS (NDDS), the drug was added into the BSA solution according to the optimized formula, and the remaining steps were consistent with the nanoparticle preparation steps. Since BSA has a negative charge and chitosan has a positive charge, the BSA nanoparticles loaded with TGF-β, IL-2 and AS are stably self-assembled with chitosan.

### 2.8 Characterization of NDDS

Scanning electron microscopy (SEM) and transmission electron microscopy (TEM) were used to observe the morphology of nanoparticles and NDDS. The average particle size of nanoparticles and NDDS was detected by Mastersizer (Malvern, England).

To detect the cytokine-released profiles of NDDS, the supernatant of NDDS was collected at 1, 2, 6, 12, 24, 48, 72, 96 and 120 ​h, respectively. The supernatant was placed in a quartz cuvette and the released rate of AS was detected at 285 ​nm by UV spectrophotometer. Meanwhile, the release curve of IL-2 (Elabscience, China) and TGF-β (MultiSciences, China) were assayed by Enzyme-Linked Immunosorbent Assay (ELISA) according to the instruction of the manufactory.

To investigate the biocompatibility of the NDDS, the CFSE-labeled lymphocytes from DBA/1 mice were cultured in the cell medium, or medium soaked in nanoparticles and NDDS. The proliferation of lymphocytes was detected on days 1, 3 and 5 by FCA.

To study the biological characters, the nanoparticles or NDDS were soaked in the complete RPMI 1640 ​cell medium, and 48 ​h later, the leaching solution was harvested. CD4^+^T cells from the DBA/1 mice sorted by magnetic beads were cultured in the leaching solution of the nanoparticles or NDDS, and the proportion of iTreg cells was determined by FCA 48 ​h later.

### 2.9 Animal procedures

CIA was generated as described previously [24]. Briefly, DAB/1 mice were injected subcutaneously into the tail of 100 μL emulsion consisting of chicken type II collagen (100 μg) mixed with an equal volume of Complete Freund's Adjuvant (CFA). And on day 21, 100 μL emulsion consisting of chicken type II collagen (100 μg) mixed with an equal volume of Incomplete Freund's Adjuvant (IFA) was injected intensively at different locations from the initial injection site. On day 28, the sera were collected and the autoantibody of IgG2c was assayed by ELISA (Chondrex, USA).

DAB/1 mice were induced into CIA. On days 28 and 33, the CIA mice were divided into four groups randomly (n ​= ​5 for each group) and injected with 20 ​μL different solutions in the joint cavity of each hind limb for intervention. The CIA-PBS group was injected with PBS, the CIA-NPs group with PBS solution containing nanoparticles, the CIA-Free group with PBS solution containing IL-2 (24.68 ​μg/mL), TGF-β (24.68 ​μg/mL) and AS (2.47 ​mM), and the CIA-NDDS group with PBS containing NDDS loaded IL-2 (35.25 ​μg/mL), TGF-β (35.25 ​μg/mL) and AS (3.41 ​mM) according to the released profiles.

### 2.10 Gross and histological evaluation of arthritis

On the 21st day after immunization, the arthritis score and body weight of DBA/1 mice were evaluated every there days. The redness and swelling of the front and rear limbs of mice were observed. Each paw was scored individually based on a 0–4 scale and the maximum score of one mouse was recorded as a score of 16 [25]. The criteria for the clinical score were summarized in the Supplementary Table 3.

On day 38, the mice were euthanatized and tissues were collected. The joints were fixed, embedded, sliced and stained with Hematoxylin-eosin (H&E) staining (Beyotime, China). In addition, Safranin-O/Fast Green (solarbio, China) was applied to stain cartilage tissues.

### 2.11 Immunological assessment

Mononuclear lymphocytes from the lymph nodes and spleens of CIA mice were isolated and the subpopulations of CD4^+^T lymphocytes, Th1, Th2, Tregs and Th17 were analyzed by FCA. At the same time, the serum TNF-α level was detected by ELISA (Elabscience, China).

### 2.12 Micro-compute tomography (Micro-CT)

Followed by the previous study [26], bone histomorphometry of the femur was analyzed by Micro-CT (SkyScan, Belgium), and the parameters were set as X-ray current of 200 ​μA, spatial resolution of 9 ​μm, filter plate 0.5 ​mm AI, X-ray voltage of 50 kv, average frame of 1, and tomographic rotation of 180°. Then, NRecon (Microphotonics, USA) and Minics (Materialise, Belgium) software were used to reconstruct the data in two and three dimensions. The bone parameters, including bone mineral density (BMD), bone volume fraction (BV/TV), trabecular spacing (Tb.Sp) and trabecular number (Tb.N) were analyzed by CTan (Bruker micro-CT, USA) software.

### 2.13 Statistical analysis

The data were denoted as mean ​± ​standard deviation (SD). Statistical analysis was performed with GraphPad Prism 7 (USA). Statistical significance was assessed by Student's t-test (two groups) or One-way ANOVA (more than two groups). The value of P ​< ​0.05 was considered to be statistically significant.

## 3 Results

### 3.1 Optimization of the IL-2/TGF-β/AS combination to induce Tregs

**Fig. 1:**
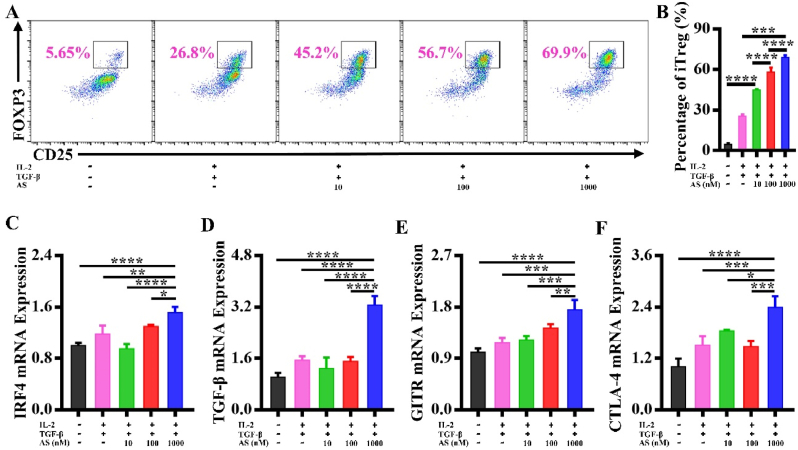
The optimal combination of IL-2/TGF-β/AS for inducing Tregs. Tregs were induced with IL-2 (10 ​ng/mL), TGF-β (10 ​ng/mL), and AS (10, 100 and 1000 ​nM, respectively). (A) Induced Tregs were detected by FCA. (B) Quantified graph of the iTregs. (C–F) Gene expression levels of IRF4, TGF-β, GITR and CTLA-4 of iTregs were analyzed by qRT-PCR, respectively. ∗*P* ​< ​0.05; ∗∗*P* ​< ​0.01, ∗∗∗*P* ​< ​0.001, ∗∗∗∗*P* ​< ​0.0001. *n* ​= ​3.

We purified CD4^+^T cells from the spleen of DBA/1 mice by magnetic beads. In order to determine the optimal combination for inducing Tregs at high efficiency, CD4^+^T cells were cultured with IL-2, TGF-β, and different concentrations of AS. Forty-eight hours later, FCA data revealed that the addition of IL-2 alone was unable to induce Tregs, while IL-2 mixed with TGF-β or AS alone was able to induce Tregs. The effect of AS combined with IL-2 and TGF-β outperformed the effect of AS alone (Fig. S1). Tregs were induced as much as 68.83 ​± ​1.37% when IL-2 (10 ​ng/mL), TGF-β (10 ​ng/mL), and AS (1000 ​nM) were combined optimally (Fig. 1A and B).

The function of Tregs is directly reflected by the expression of IRF4, TGF-β, GITR, and CTLA-4 in Tregs [[27], [28], [29]]. As a result, qRT-PCR data demonstrated that, considerably different from the other groups, the optimized combination of IL-2/TGF-β/AS, could enhance the gene expression of IRF4, TGF-β, GITR, and CTLA-4 in the iTregs at most (Fig. 1C–F).

### 3.2 Immunosuppression of Tregs induced by the optimized combination of IL-2/TGF-β/AS

**Fig. 2:**
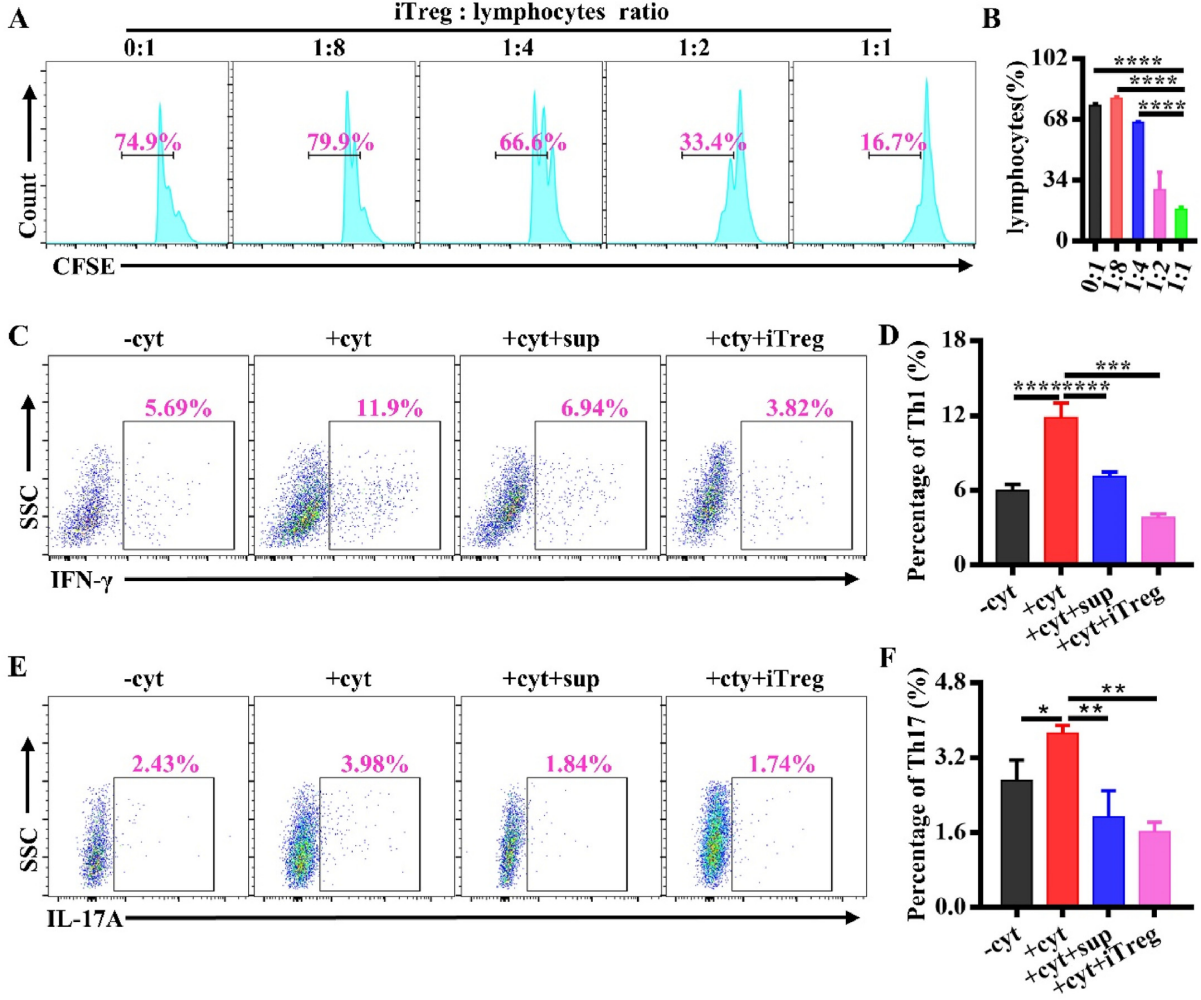
Immunosuppression of Tregs induced by the optimized combination of IL-2/TGF-β/AS. (A) CFSE-stained lymphocytes isolated from BALB/c mouse were co-culture with the iTregs at the different ratios for 72 ​h and analyzed by FCA (iTreg: Lymphocyte ​= ​0:1, 1:8, 1:4, 1:2; 1:1, respectively). (B) Quantification of CFSE staining of lymphocytes. (C) Lymphocytes were co-cultured with the iTregs at 1:1 or the culture supernatant of the iTregs, in the presence of IL-2, IL-6, IL-12 and TGF-β. The frequency of Th1 (CD4^+^IFN-γ^+^) was detected by FCA. (D) The percentage of Th1 in total lymphocytes. (E) Lymphocytes were co-cultured with the iTregs at the ratio of 1:1 or the culture supernatant of the iTregs, in the presence of IL-2, IL-6, IL-12 and TGF-β. The frequency of Th17 (CD4^+^IL-17A^+^) was detected by FCA. (F) The percentage of Th17 in total lymphocytes. - cyt: lymphocytes; + cyt: lymphocytes in the presence of IL-2, IL-6, IL-12 and TGF-β; +cyt ​+ ​sup: lymphocytes in the presence of IL-2, IL-6, IL-12, TGF-β and the supernatant of the iTregs; +cyt ​+ ​iTreg: lymphocytes in the presence of IL-2, IL-6, IL-12, TGF-β and the iTregs. ∗*P* ​< ​0.05; ∗∗*P* ​< ​0.01, ∗∗∗*P* ​< ​0.001, ∗∗∗∗*P* ​< ​0.0001. *n* ​= ​3.

To assess whether the Tregs induced by IL-2/TGF-β/AS has the capacity to impede T cell growth, lymphocytes from the spleen of BALB/c mouse were cultured with the iTregs at a variety of ratios. As seen in Fig. 2A and B, compared with the lymphocytes alone, the proliferation of lymphocytes in the presence of the iTregs was lower. Of interest, the inhibition of proliferation was stronger and stronger, as the frequency of the iTregs increased, suggesting a dose-depended inhibitory effect of the iTregs.

Tregs play an immunosuppressed role either by direct cell-cell interaction or paracrine, such as the secretion of IL-10, interleukin-4 (IL-4) and TGF-β [30]. Therefore, we simulated the inflammatory environment by adding cytokines of interleukin-12 (IL-12), IL-2, interleukin-6 (IL-6) and TGF-β in the cell medium to differentiate CD4^+^T cells into Th1 and Th17 phenotypes [31]. Meanwhile, we co-cultured lymphocytes with the iTregs or the culture supernatant (collected from the iTrges 24hr after deprivation of IL-2/TGF-β/AS). The FCA data demonstrated that the cytokines successfully induced the differentiation of Th1 (marked as CD4^+^IFN-γ^+^) and Th17 (marked as CD4^+^IL-17A^+^) cells. However, upon the addition of the iTregs or the culture supernatants, the frequencies of Th1 and Th17 ​cells dropped significantly (Fig. 2C–F). In a word, the Tregs induced by the optimized IL-2/TGF-β/AS combination, retained their immunosuppressed ability and inhibited the differentiation of Th1 and Th17 ​cells through paracrine or cell contact, even in an inflammatory environment.

### 3.3 The optimal combination of IL-2/TGF-β/AS induced Treg cells with CIA

**Fig. 3:**
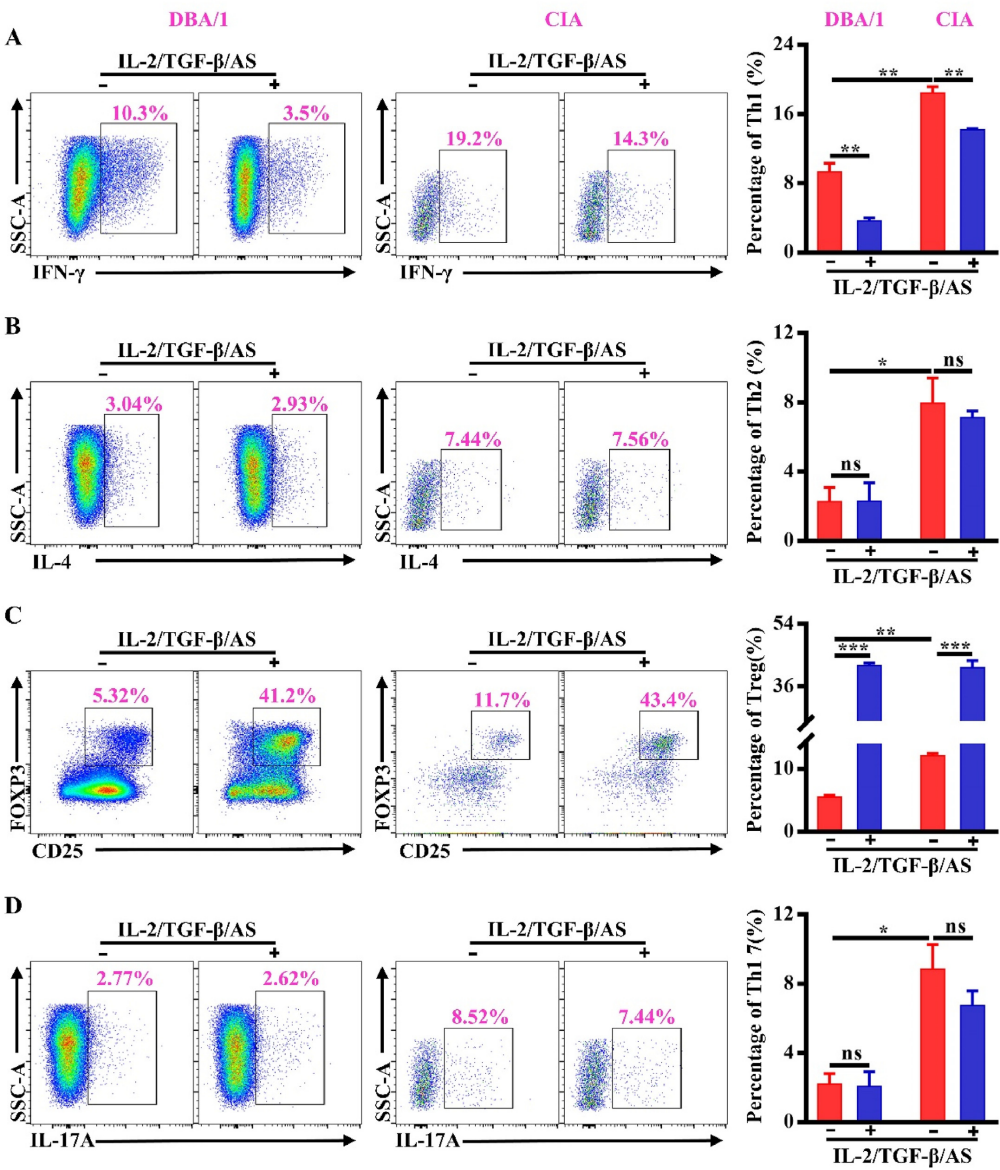
The IL-2/TGF-β/AS combination induced Treg cells in the DBA mice with and without CIA. (A) The proportion of Th1 (CD4^+^IFN-γ^+^) cells differentiated from the splenocytes of control mice and CIA mice were detected by FCA. (B) The proportion of Th2 (CD4^+^IL-4^+^) cells differentiated from the splenocytes of control mice and CIA mice was detected by FCA. (C) The proportion of iTreg (CD4^+^CD25^+^Foxp3^+^) cells differentiated from the lymphocytes of control mice and CIA mice were detected by FCA. (D) The proportion of Th17 (CD4^+^IL-17A^+^) cells differentiated from the lymphocytes of control mice and CIA mice were detected by FCA. Control: DBA mice, CIA: DBA mice with CIA. ∗*P* ​< ​0.05; ∗∗*P* ​< ​0.01, ∗∗∗*P* ​< ​0.001, ns ​= ​not significant. *n* ​= ​5.

We further explore the effect of the optimal combination of IL-2/TGF-β/AS on inducing Tregs from the DBA/1 mouse splenocytes with CIA. CD4^+^T cells from the DBA/1 mice 38 days after initial immunization and treated with IL-2/TGF-β/AS. Compared with normal DBA/1 mice, the proportions of Th1, Th2, iTreg, and Th17 ​cells in CIA mice were higher than those in normal mice (Fig. 3). The ratio of Th1/Th2 and Treg/Th17 in the CIA mice was different from the ones in the normal mice, indicating that the mice were in an inflammatory state after collagen priming.

In normal conditions, with the addition of IL-2/TGF-β/AS, the frequency of Th1 decreased by around 70% and iTregs increased around eight times, compared to the ones without cytokine stimulation (Fig. 3A and C). However, the frequency of Th2 and Th17 was not significantly changed (Fig. 3B and D). As expected, in the CIA status, treated with IL-2/TGF-β/AS, the change of Th1 and iTregs proportions showed the same trend as the ones in normal conditions. But, the cytokine-mediated decrease ratio of Th1 from the CIA mice was less than that of the normal mice. Impressively, the cytokine-mediated increase ratio of iTregs from the CIA mice was similar to that of the normal mice. (Fig. 3). In sum, the potential of the optimal combination of IL-2/TGF-β/AS to induce Tregs was not influenced by the microenvironment, neither physiological nor inflammatory condition.

### 3.4 Characterization of NDDS

**Fig. 4:**
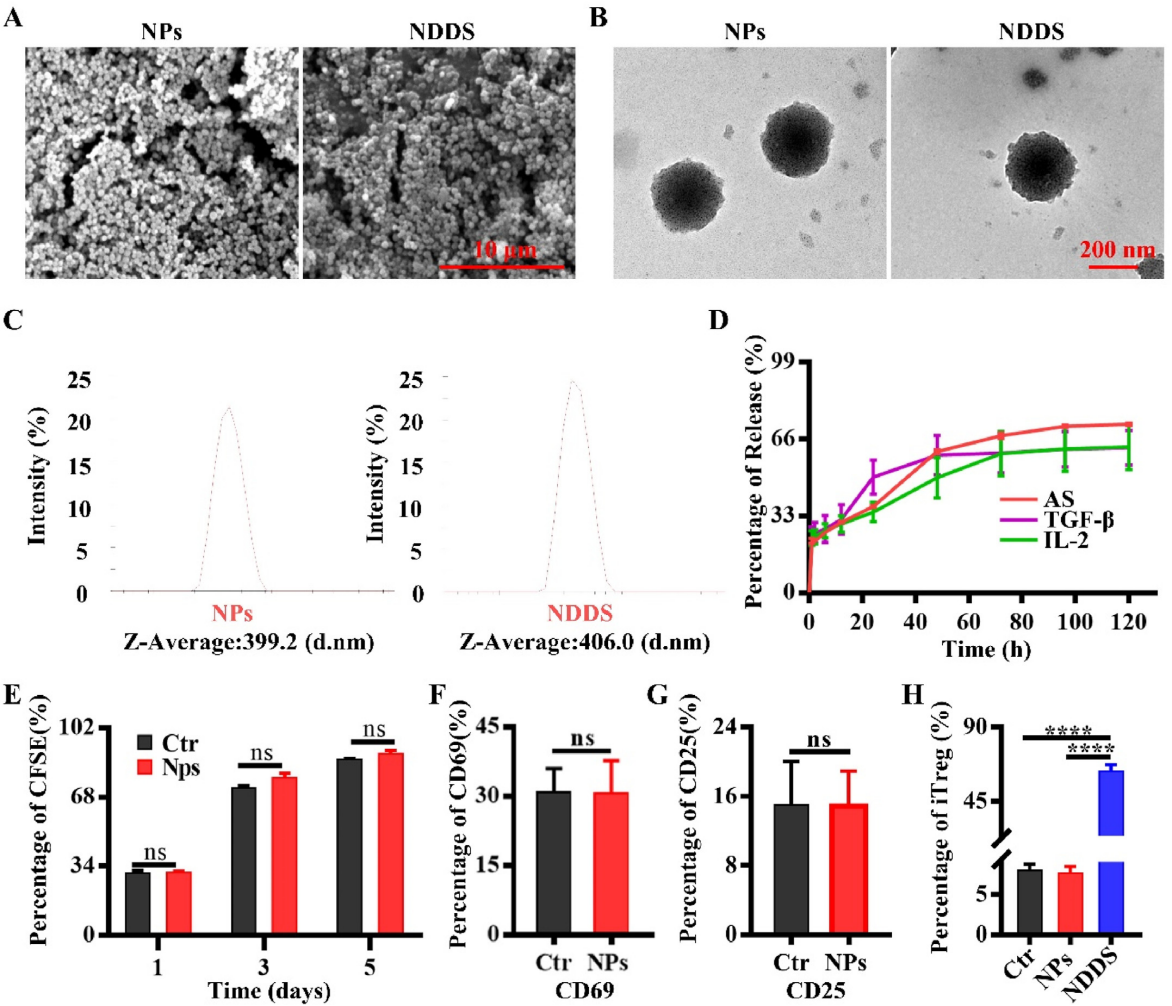
Characterization of NPs and NDDS. (A) Representative SEM images of NPs and NDDS. (B) Representative TEM images of NPs and NDDS. (C) The particle size distribution of NPs and NDDS. (D) The release curve of TGF-β, IL-2 and AS of NDDS. (E) The proliferation of lymphocytes was stained with CFSE and detected by FCA on day 1, 3, and 5. (F) CD69 expression in CD3^+^T cells was analyzed by FCA. (G) CD25 expression in CD3^+^T cells was analyzed by FCA. (H) The frequency of iTreg cells was detected by FCA. Ctr: cell culture medium, NPs: cell culture medium soaked in NPs, NDDS: cell culture medium soaked in NDDS. ∗∗∗∗*P* ​< ​0.0001, ns ​= ​not significant. *n* ​= ​3.

We prepared nanoparticles (NPs) and a chitosan-stabilized nanoparticle drug delivery system with IL-2/TGF-β/AS (NDDS) by desolvation technique following the published paper [32]. Their morphological structures were studied by SEM and TEM, respectively. NPs and NDDS were both scattered, uniform in size, and exhibited no discernible differences in their morphology or structure (Fig. 4A and B). The Z-Average particle sizes of NPs and NDDS were, respectively, 399.2 ​nm and 406 ​nm, determined by the Malvern particle size analyzer (Fig. 4C).

We explored the released profiles of the cytokines by measuring the amounts of the cytokines in the supernatant at the indicative times. The release rates of AS, IL-2, and TGF-β at 96 ​h were 71.48 ​± ​0.41%, 61.77 ​± ​7.85%, and 61.59 ​± ​6.26%, respectively, as shown in Fig. 4D. The released profile demonstrated that nanoparticles released drugs sustainably in a certain time.

Then we cultured lymphocytes in the leachate of the NPs and investigated the proliferation of cells. The data showed that there was no significant difference in lymphocytes proliferation between the different groups (Fig. 4E). Additionally, there was no discernible difference between the early activation parameter CD69 (Fig. 4F) and the late activation parameter CD25 of T cells, showing that NPs had good biocompatibility (Fig. 4G).

Of expectation, we treated CD4^+^T cells isolated from the splenocytes of DBA/1 mice with NPs or NDDS leaching solutions, and the results revealed that the differentiation ratio of iTreg cells generated by the two solutions was 7.59 ​± ​0.71% and 63.23 ​± ​3.31%, respectively (Fig.4H, Fig. S2). This suggests that NDDS is capable of biological activity to induce Tregs.

### 3.5 NDDS alleviated collagen-induced arthritis in DBA/1 mice

**Fig. 5:**
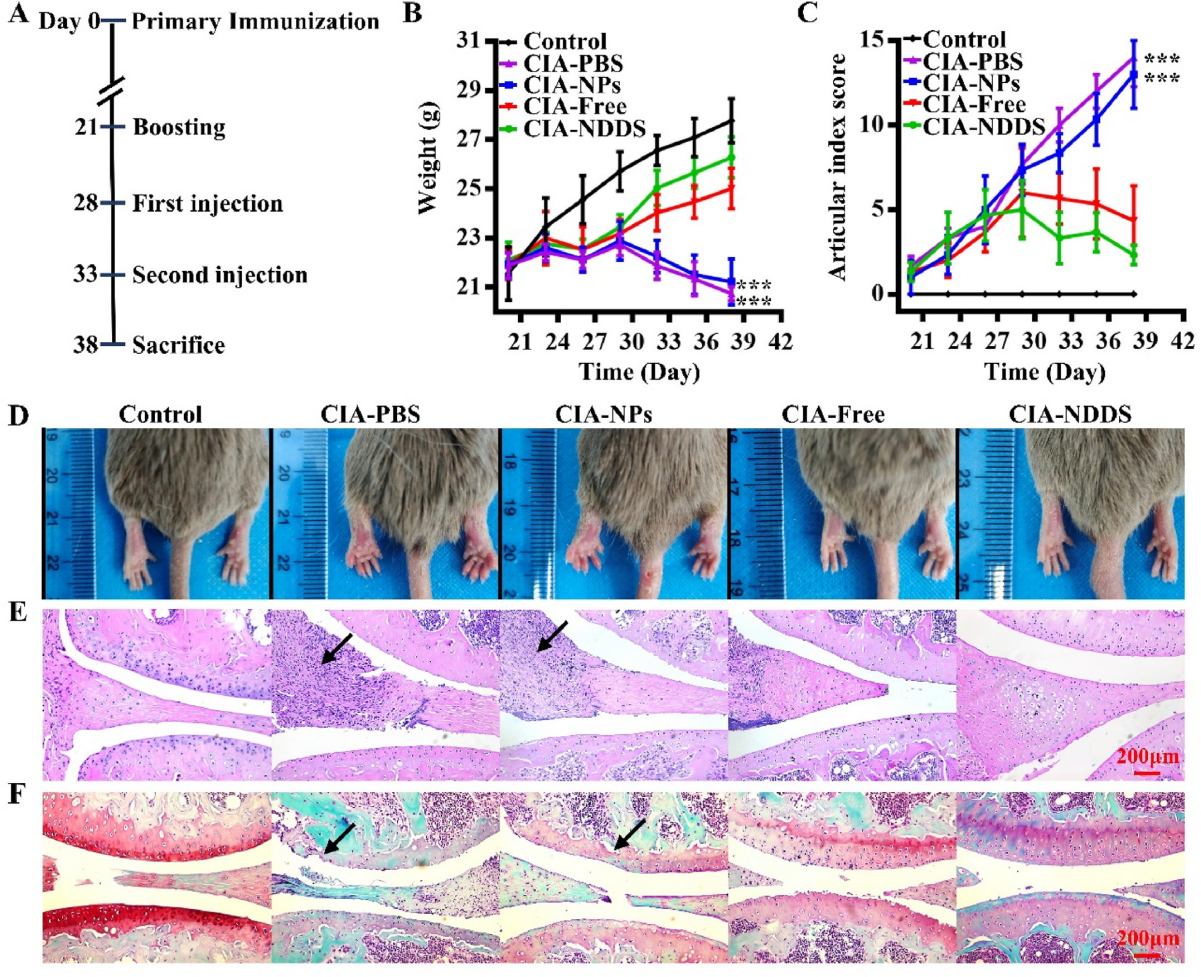
NDDS alleviated collagen-induced arthritis in DBA/1 mice. (A) Experiment flow chart. (B) Body weight of the different groups at different time points. Comparing CIA-NDDS and three other samples at the same time point via one-way analysis of variance (ANOVA). ∗∗∗*P* ​< ​0.001. *n* ​= ​5. (C) Scoring of the articular index of the different groups. Comparing CIA-NDDS and three other samples at the same time point via one-way analysis of variance (ANOVA). ∗∗∗*P* ​< ​0.001. *n* ​= ​5 (D) Macroscopic observations of the redness and swelling of toes 38 days after the first immunization. (E) H&E staining of representative knee sections (black arrows represented the inflammatory cell infiltration). (F) Safranin-O/Fast Green staining of representative knee sections (black arrows represented the cartilage degradation). Control: normal DBA/1 mice, CIA-PBS: CIA mice injected with PBS, CIA-NPs: CIA mice injected with NPs, CIA-Free: CIA mice injected with free cytokines, CIA-NDDS: CIA mice injected with NDDS.

The CIA was induced successfully by immunization of DBA/1 mice on days 0 and 21 (Fig. S3). NDDS (the NDDS group) were administrated on days 28 and 33, according to the cytokine released prolife of NDDS. Meanwhile some of the CIA mice were treated with vehicle PBS (the CIA group), NPs (the NPs group) or the same dose of free cytokine composition (the free group) as NDDS in the same volume. Mice were euthanized on day 38 and samples were collected. This pipeline of experimental procedures was depicted in Fig. 5A. Interestingly, the body weight, an indicator of RA, was affected by the initiation of CIA. Compared with the body weight of the normal DBA mice, the ones of CIA mice with different treatments dropped with varying degree. However, NDDS and free cytokine-treated group greatly minimized their weight loss caused by RA (Fig. 5B). NDDS rescued more body weight from a loss than free cytokines did.

The joint score scale is one of the indications for the progression of the disease and the function of the joints [33]. The higher the score, the more serious the RA is. Compared with the normal mice, the joint score of the CIA mice dramatically increased (Fig. 5C). However, with different treatments, the joint score of the NDDS group had the lowest score. Impressively, the joint score of CIA mice treated with free cytokine composition was lower than the one of the NPs group but still higher than the one of the NDDS group (Fig. 5C).

When CIA was successfully induced in DBA/1 mice, the joints displayed redness, swelling, and immobility. Redness and swelling were faded when the intervention was performed (Fig. 5D). The knee joint was stained with H&E and Safranin-O/Fast Green, respectively. The arthritis of CIA mice from both the Free and NDDS group reduced inflammatory cell infiltration, synovial hyperplasia, and cartilage degradation (Fig. 5E and F), but the images showed a better outcome in the NDDS than in the Free group. Overall, the systematic evidence showed that NDDS had a significant therapeutic effect on alleviating arthritis-related inflammation and disease in the CIA mice.

### 3.6 NDDS protected subchondral bone mass from loss in the CIA mice

**Fig. 6:**
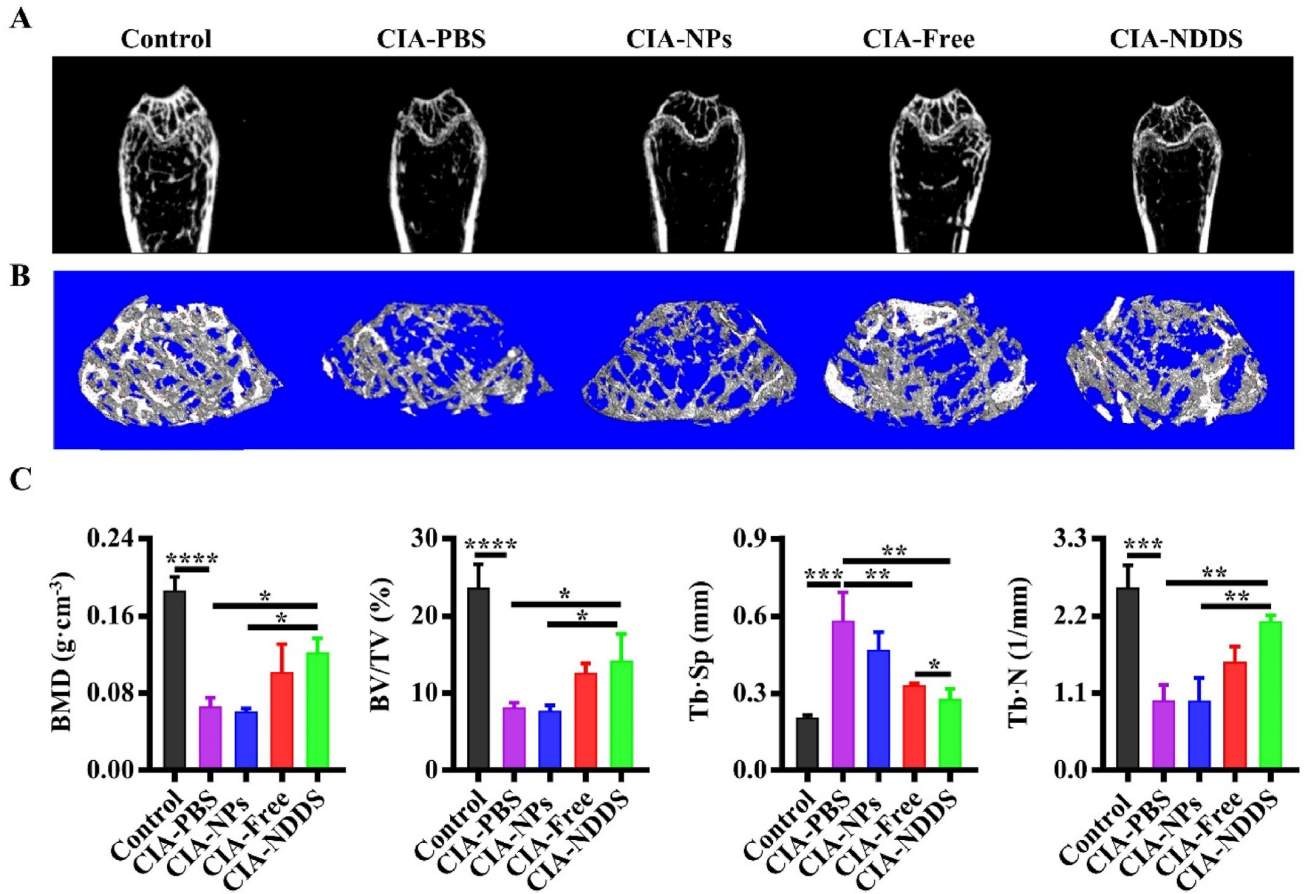
NDDS protected subchondral bone mass from loss in the CIA mice. (A) 2D reconstructed images of the trabecular femur of the mice. (B) 3D reconstructed images of the trabecular femur of the mice. (C) Quantitative analysis of bone-related parameters by Micro-CT scanning. BMD: Bone mineral density, BV/TV: Bone volume fraction, Tb. Sp: Trabecular spacing, Tb.N: Trabecular number. Control: normal DBA/1 mice, CIA-PBS: CIA mice injected with PBS, CIA-NPs: CIA mice injected with NPs, CIA-Free: CIA mice injected with free cytokines, CIA-NDDS: CIA mice injected with NDDS. ∗*P* ​< ​0.05; ∗∗*P* ​< ​0.01, ∗∗∗*P* ​< ​0.001, ∗∗∗∗*P* ​< ​0.0001. *n* ​= ​5.

The subchondral bone mass loss often happens in CIA [34]. It was noted that the CIA and NPs groups had statistically lower bone mass than the Free and NDDS groups (Fig. 6A and B). In comparison to the ones of the CIA and NPs groups, the BMD and TB. Sp parameters of the NDDS group were significantly higher. On the contrary, The BV/TV and TB. N parameters of the CIA and NPs group were significantly lower than that of the NDDS group (Fig. 6C). Thus, the delivery of NDDS protected subchondral bone mass from loss in the CIA mice.

### 3.7 NDDS reduced inflammation in the CIA mice

**Fig. 7:**
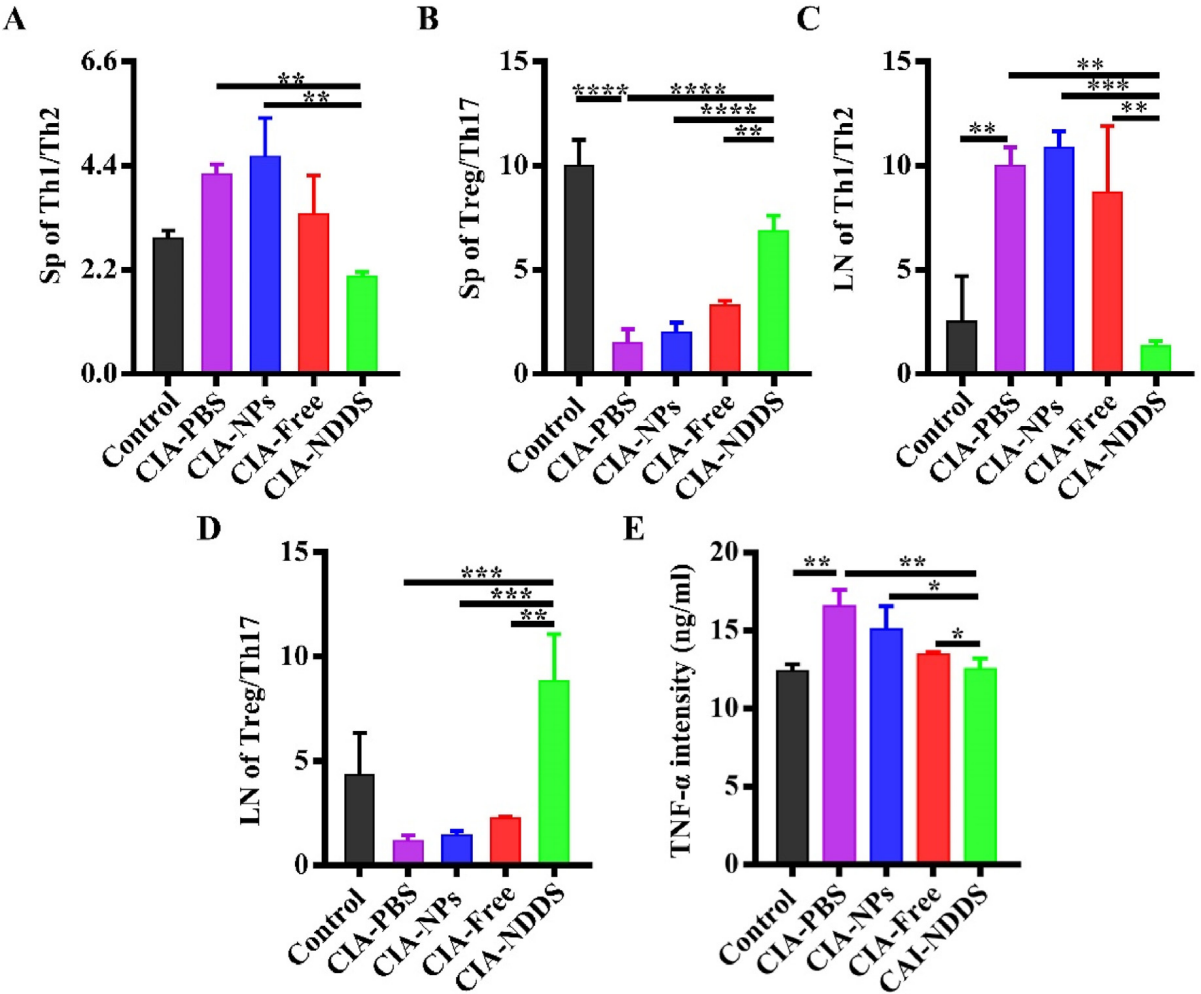
NDDS reduced inflammation in the CIA mice. (A) The ratio of Th1/Th2 cells in the spleen. (B) The ratio of Treg/Th17 ​cells in the spleen. (C) The ratio of Th1/Th2 cells in the Lymph node. (D) The ratio of Treg/Th17 subset cells in the Lymph node. (E) Serum TNF-α levels. Control: normal DBA/1 mice, CIA-PBS: CIA mice injected with PBS, CIA-NPs: CIA mice injected with NPs, CIA-Free: CIA mice injected with free cytokines, CIA-NDDS: CIA mice injected with NDDS. ∗*P* ​< ​0.05; ∗∗*P* ​< ​0.01, ∗∗∗*P* ​< ​0.001, ∗∗∗∗*P* ​< ​0.0001. *n* ​= ​5.

TNF-α has a significant function in mediating arthritic damage and may exacerbate the severity and course of RA illness [35]. CIA mice treated with NDDS had significantly lower TNF-α secretion in the sera than the other CIA-induced groups (Fig. 7A).

The joint deterioration in CIA mice may eventually result from immune system malfunction and invading effector cells. By FCA, the subtype population of CD4^+^T cells in the spleen and lymph nodes was analyzed. As found in Fig. 7B–E, compared with the normal mice, the CIA mice had a higher ratio of Th1/Th2 and a lower ratio of Treg/Th17, respectively, either in the spleens or lymph nodes, which suggested that the CIA mice were in an inflammation status. NDDS injection could dramatically decrease Th1 and Th17 ​cell frequency in CIA mice and reverse the ratio of Th1/Th2 and Treg/Th17 ​cells, respectively, because of the increased number of iTregs (Fig. S4). It demonstrated that, with the administration of NDDS, the ratio of Th1/Th2 and Treg/Th17 in the CIA mice recovered from misbalance at some degree, and the inflammation of the CIA mice was also reduced consequently.

## 4 Discussion

Generally, immune tolerance is a complex dynamic process that involves a coordinated network of immune cells and molecules. It is regulated by pro-inflammatory and anti-inflammatory factors in the microenvironment, such as Th1/Th2, Treg/Th17, which are usually in a balance in the physiological condition. If pro-inflammatory factors are predominant, the balance is disturbed and autoimmune diseases develop. Thus, creating a favorable environment for the physiologically dynamic immunological balance is a promising strategy for treating autoimmune diseases.

Recently, the research on the regulation of Treg/Th17 ​cell balance has drawn a great amount of sight since the ratio of Treg/Th17 ​cells shifting to Tregs is favorable to treat autoimmune diseases [36,37]. Thus, Treg-target inducible platform with high efficiency is of great significance in the clinics. In this study, we first optimized a pharmacological cocktail of IL-2, TGF-β, and AS to induce Treg cells high-efficiently through continuous screening. After induction, this optimized combination boosted the proportion of iTreg cells to 68.83 ​± ​1.37%, which was much higher than either IL-2/TGF-β or as alone did. AS can promote Foxp3 expression in T cells by activating janus kinase (JAK)/STAT5 pathway, which starts active transcription in Treg cells. And IL-2 can directly and competitively inhibit STAT3 through STAT5, downregulate the expression of RORγt, and enhance the expression of FOXP3 induced by TGF-β. Foxp3 promoter and enhancer binding by activated STAT5 triggers active transcription in Treg cells and tilts CD4^+^T cell differentiation in favor of Tregs. Our *vitro* results displayed that Tregs induced by IL-2/TGF-β/AS had an immunosuppressive function both in the physiological and inflammatory environment, which is consistent with relevant studies [15]. Thus, the optimized drug combination provides an ideal formula for solving the problems of low induction efficiency, and provides an experimental basis for iTreg cells in the treatment of autoimmune diseases.

Released burst and rapid diffusion are other concern when free drugs are delivered *in vivo* directly. With the help of biomaterials, sustainable and controlled release of drugs becomes possible [38]. Research has confirmed that albumin is an excellent vehicle to deliver drugs to treat disease [39]. Fan N et al. loaded celastrol into BSA nanoparticles and produced Celastrol-BSA-NPs by high-pressure homogenization technology, which can reduce lipid accumulation and improve insulin sensitivity to treat diet-induced obesity [40]. Studies have shown that albumin can be prepared into nanoparticles for drug delivery by desolvation technique, emulsification, self-assembly and nanoparticle albumin-bound (NAB) technology [[41], [42], [43]]. However, the emulsification method is easy to destroy the stability of albumin and has poor repeatability, and the self-assembly method will leave toxic β-mercaptoethanol or dithiothreitol and other reducing agents. NAB technology has higher requirements for equipment, and may cause the problem of difficult dispersion of lyophilized products due to the aggregation of albumin in the homogenization process. Therefore, we use the solvent removal method to make a nano-scale carrier because of its simple operation, such as fewer preparation steps, fast reaction speed, no need for special equipment, and so on.

In this study, we constructed NDDS by means of the desolvation technique following the previous research [32,44], and mixed the optimal combination of IL-2/TGF-β/AS with BSA solution evenly. With adding absolute ethanol, negatively charged BSA nanoparticles were precipitated, and then positively charged chitosan was added. The thin shell of chitosan restricts the swelling and self-adhesion of BSA nanoparticles. Due to its electrostatic self-assembly, stably drug-loaded nanoparticles were finally formed. Therefore, NDDS can make the drug release slowly, prolong the biological half-life of the drug, avoid released burst and rapid diffusion. *In vitro* experiments, we found that the chitosan-stabilized BSA vector was homogeneous in size, had good biocompatibility, and had no effect on the activation and proliferation of T cells.

Compared with the normal DAB/1 mice, we found that the imbalance of Th1/Th2 and Treg/Th17 existed in the mice with CIA, which contributed to the inflammatory environment of RA. Consequently, the inflammatory environment attributed to degrade chondrocytes and destroy subchondral bone microstructure, which was evidenced in our study. Notably, after NDDS injection, we found that NDDS better alleviated the lesions of CIA in mice as well as bone loss, compared with other groups. It may be because the sustained IL-2/TGF-β/AS release increased the proportion of Tregs dramatically and decreased the proportion of Th1 cells, Th17 ​cells and secretion of TNF-α, thereby shifting the imbalance of Th1/Th2 and Treg/Th17 to a less inflammatory phenotype. Thus, NDDS displays a promising therapeutic effect in RA.

However, there are some limitations in our study. We have only explored the effect of this combination of IL-2/TGF-β/AS on CD4^+^T subtype cells. Other immune cells, such as CD8^+^T subpopulation cells, are also needed to be further investigated. Meanwhile, in order to enhance NDDS’ therapeutic effect and minimize the side effects of drugs, we need to make a more detailed exploration on the frequency and dose of drug administration with biomaterials.

## 5 Conclusion

In the present study, we screened out the optimal combination of IL-2, TGF-β, and AS that high-effectively induced iTreg cells. We further constructed NDDS and injected them locally into the knee joint to alleviate the disease of CIA in mice. Mechanically, the NDDA administration promoted Treg differentiation, consequently restored the equilibrium of Treg/Th17, and decreased the secretion of TNF-α in the sera. This study provides a new immunotherapy strategy for RA as well as other autoimmune diseases, but the deep biological mechanism and clinical translation need to be further studied.

## Credit author statement

**Lin Wang:** Conceptualization, Methodology, Writing- Original Draft, Data Curation, Investigation. **Yi Wang:** Methodology, Data Curation, Software. **Chang Liu:** Data Curation, Formal analysis. **Jiachen He:** Data Curation, Validation, Formal analysis. **Xu He:** Data Curation, Validation. **Xiongjinfu Zhang:** Data Curation. **Can Zhu:** Data Curation, Validation. **Jie Sun:** Conceptualization, Project administration, Supervision. **Qin Wang:** Project administration, Supervision, Resources. **Hao Chen:** Project administration, Supervision. **Qin Shi:** Methodology, Conceptualization, Resources, Writing- Review & Editing, Project administration, Supervision.

## Declaration of competing interest

The authors declare that they have no known competing financial interests or personal relationships that could have appeared to influence the work reported in this paper.

## Data Availability

Data will be made available on request.
