# Supplementary material for: Treg-targeted efficient-inducible platform for collagen-induced arthritis treatment
Source: Mater Today Bio. 2023 Jan 20;19:100557. doi: 10.1016/j.mtbio.2023.100557 (PMC9874074; doi:10.1016/j.mtbio.2023.100557)
Supplement: Multimedia component 1 [file mmc1.docx]

**Supplementary Materials**

**Treg-targeted efficient-inducible platform for collagen-induced arthritis treatment**

Lin Wang^a,*^, Yi Wang^a,*^, Chang Liu^a *^, Jiachen He^a^, Xu He^a^, Xiongjinfu Zhang^a^, Can Zhu^a^, Jie Sun ^a, †^ , Qin Wang^b, †^, Hao Chen^c, †^, Qin Shi^a,d^ ^†^

^a^ Department of Orthopedics, the First Affiliated Hospital of Soochow University, Orthopedic Institute of Soochow University, Suzhou Medical College of Soochow University, 899 Pinghai Road, Suzhou, Jiangsu, 215031, P.R. China

^b^ Department of Immunology, School of Biology & Basic Medical Sciences, Suzhou Medical College of Soochow University, 199 Renai Road, Suzhou, Jiangsu, 215021, P.R. China

^c^ Department of Orthopedics, Affiliated Hospital of Yangzhou University, Yangzhou University, No. 368, Hanjiang middle Rd, Yangzhou, Jiangsu, 225000, P.R. China

^d^ Department of Orthopedics, Wuxi Ninth People’s Hospital affiliated to Soochow University, Wuxi, Jiangsu, 214026, P. R. China.

*These authors contributed equally to this work

†Corresponding Author(s): shiqin@suda.edu.cn (Qin Shi), [hchen2020@yzu.edu.cn](mailto:hchen2020@yzu.edu.cn) (Hao Chen), [wangqin78@suda.edu.cn](mailto:wangqin78@suda.edu.cn) (Qin Wang), [sunjie0829@suda.edu.cn](mailto:sunjie0829@suda.edu.cn) (Jie Sun)


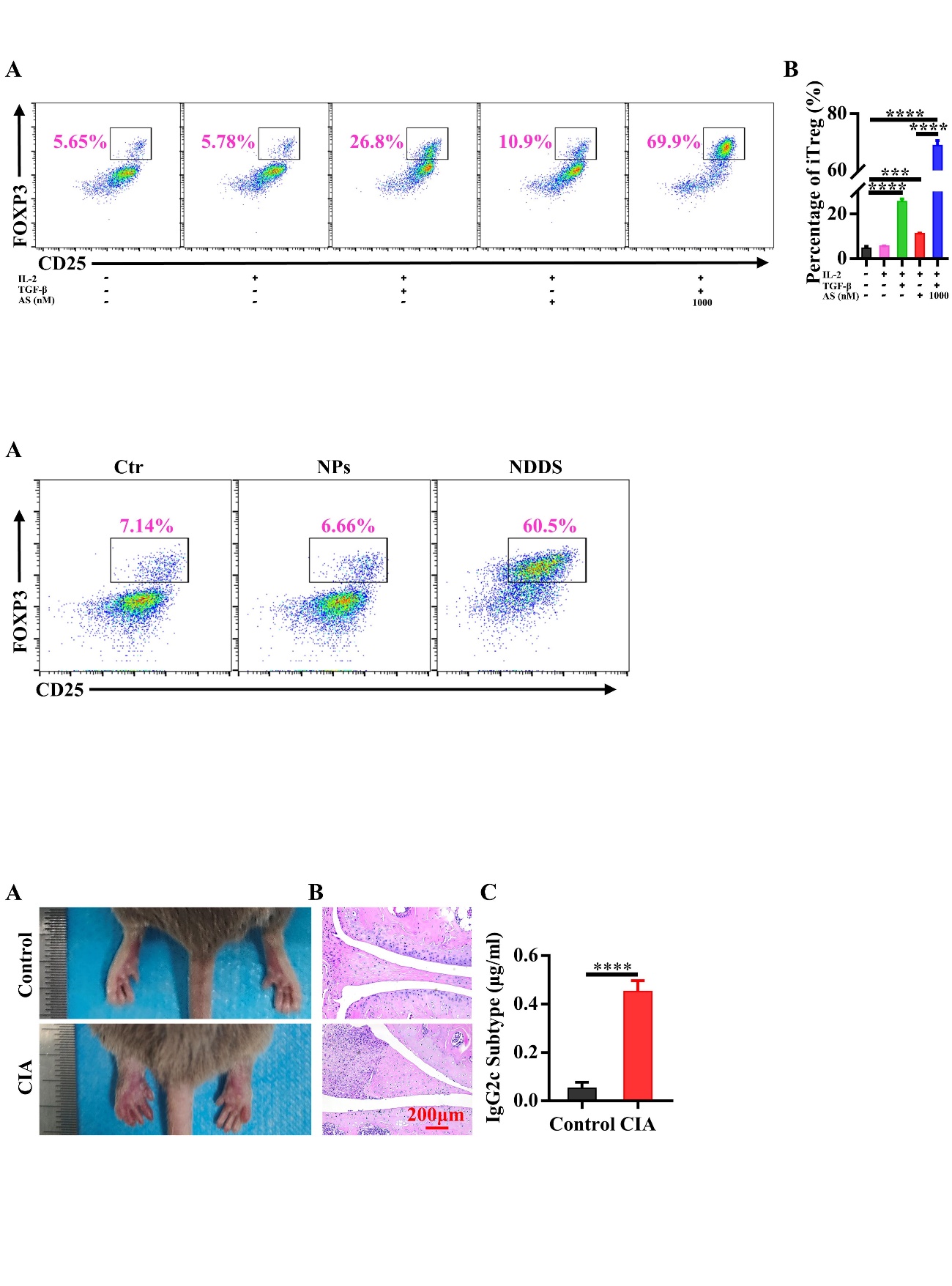


**Fig.S1** Tregs were induced by IL-2 (10 ng/mL), TGF-β (10 ng/mL) and different combinations of AS (10, 100, 1000 nM), respectively. (A) The frequency of iTregs were detected by FCA. (B) Quantified graph of the frequency of iTregs. ****P*< 0.001, *****P*< 0.0001. *n* = 3.


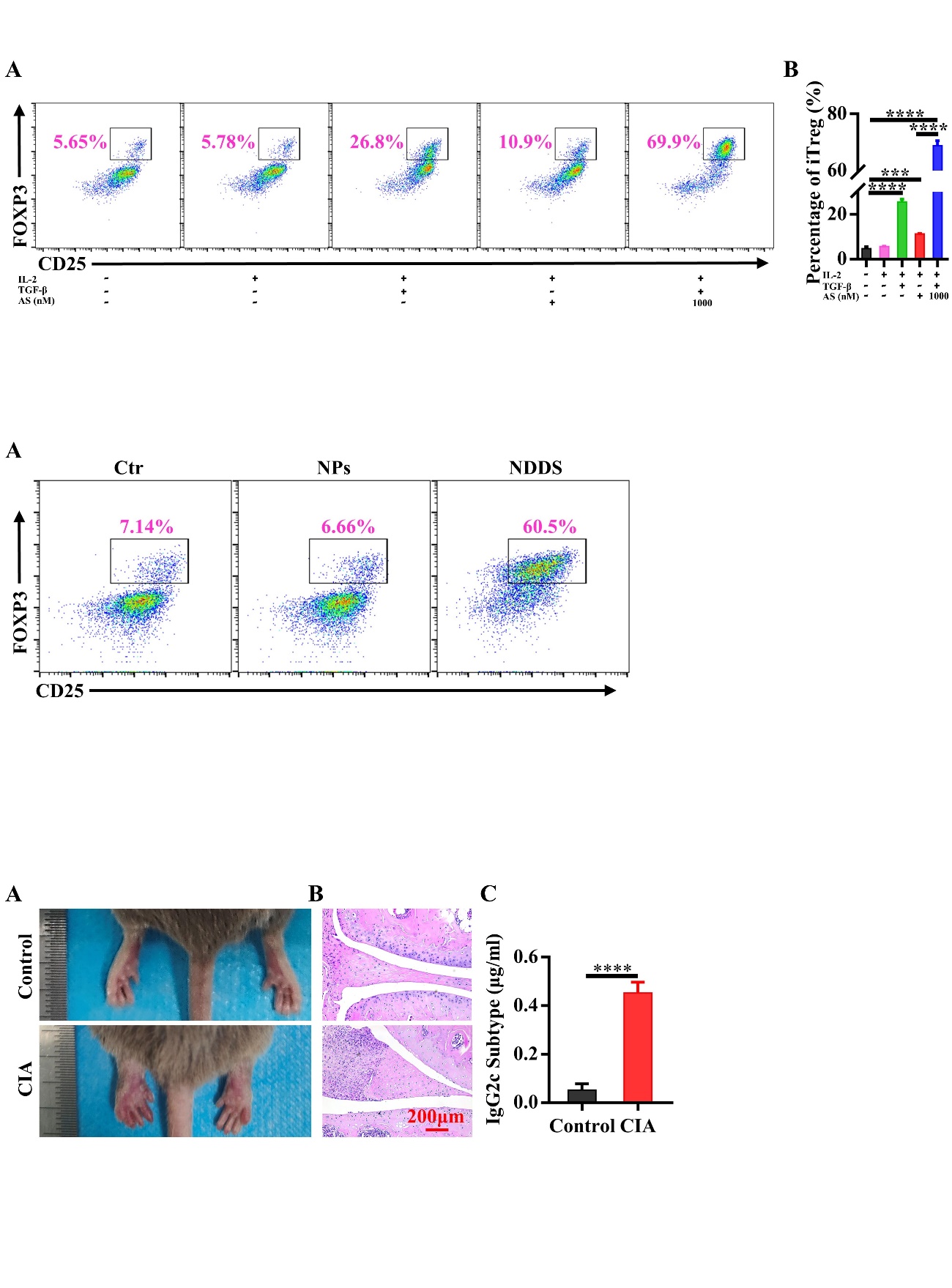


**Fig.S2** Tregs was induced by the leachate solution of nanoparticles and NDDS. (A) The frequency of iTregs were detected by FCA. Ctr: complete cell culture medium, NPs: the leachate solution of NPs, NDDS: the leachate solution of NDDS.


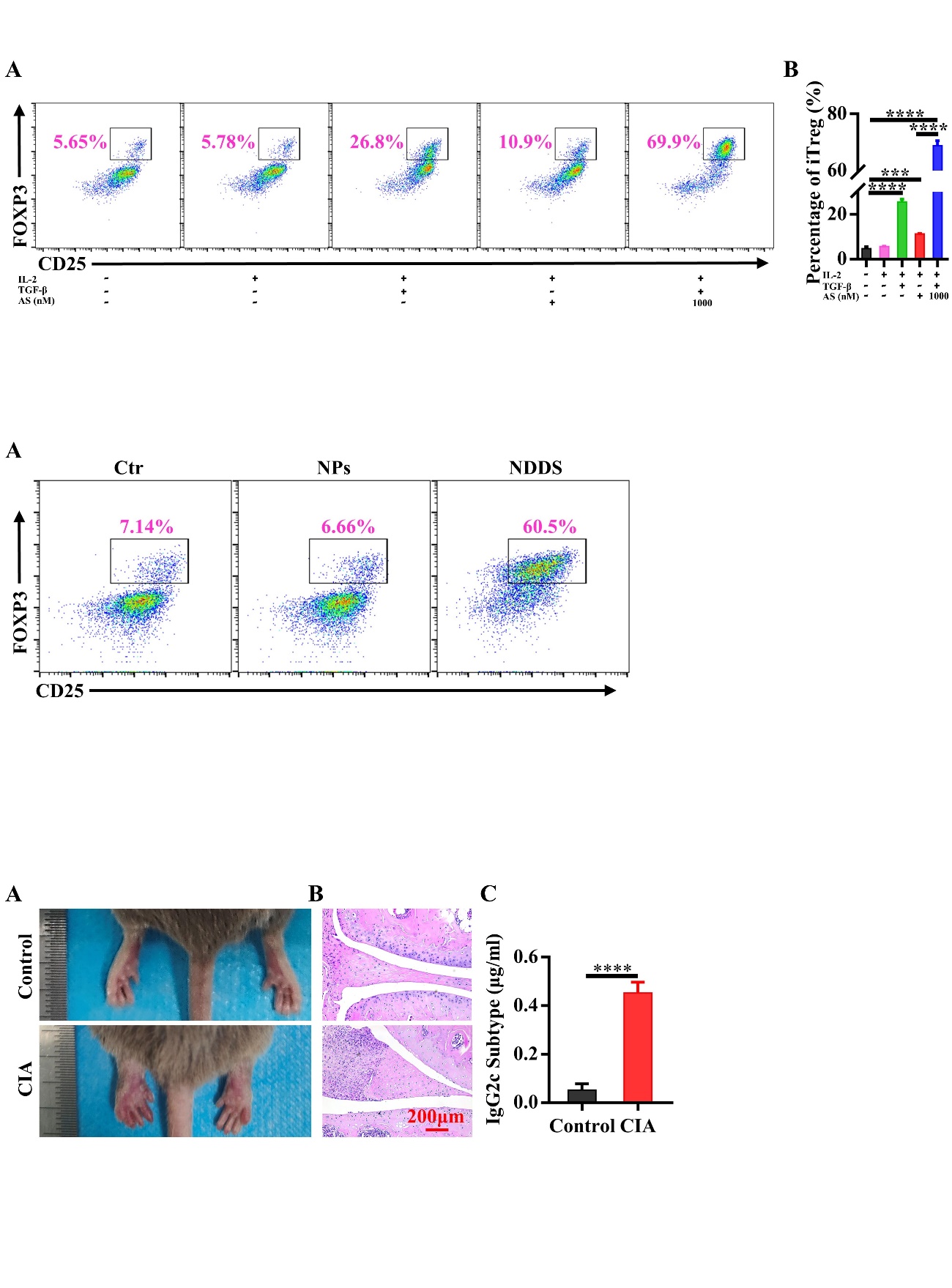


**Fig.S3** CIA were induced in the DBA/1 mice. (A) Macroscopic observations of the redness and swelling of toes, 28 days after the first immunization. (B) Haematoxylin and eosin (H&E) staining of knee joint on day 28. (C) The sera were collected on day 28 and autoantibody of IgG2c were assayed by ELISA kit. Ctr: normal DAB/1 mice, CIA: CIA-modeled DAB/1 mice. *****P*< 0.0001. n=5

**
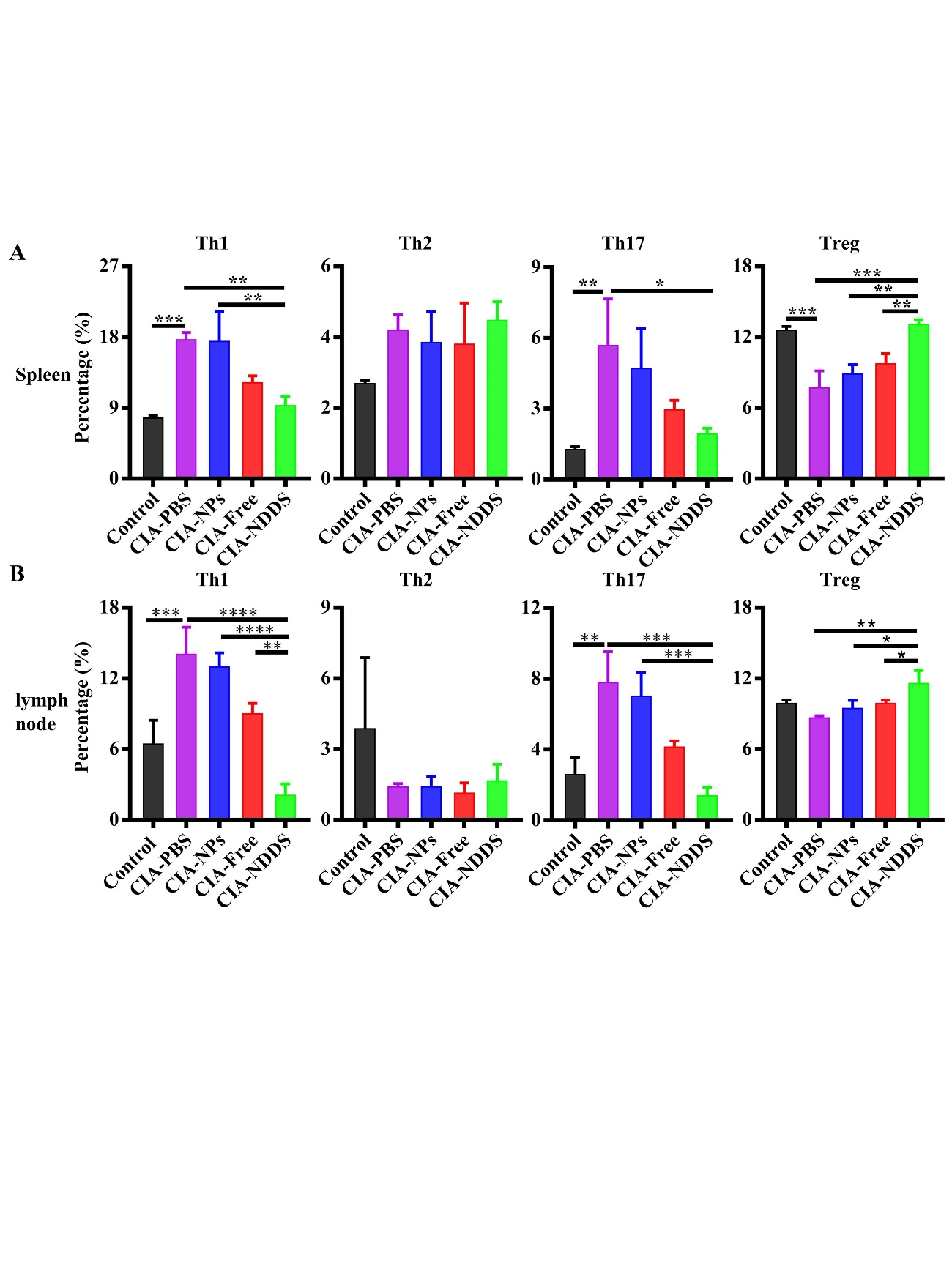
**

**Fig.S4** NDDS reduced inflammation in the CIA mice. The frequency of Th1, Th2, iTreg and Th17 cells from the spleens and lymph nodes were detected by FCA on day 38 after the immunization. (A) The splenocytes of the mice (B) The cells from the lymph node of the mice. Control: normal DBA/1 mice, CIA-PBS: CIA mice injected with PBS, CIA-NPs: CIA mice injected with NPs, CIA-Free: CIA mice injected with free cytokines, CIA-NDDS: CIA mice injected with NDDS. **p* < 0.05; ***p* < 0.01, ****P*< 0.001, *****P*< 0.0001. n=5

**Table S1. Reagents and antibodies**

| **Reagents** | **Vendor** | **Catalog**  **number** |
| --- | --- | --- |
| Type II collagen (CII) | Chondrex | 20012 |
| complete Freund’s Adjuvant (CFA) | Chondrex | 7008 |
| incomplete Freund’s adjuvant (IFA) | Chondrex | 7002 |
| Ultra-LEAF™ purified anti-mouse CD3ε Antibody | Biolegend | 100339 |
| Ultra-LEAF™ purified anti-mouse CD28 Antibody | Biolegend | 102115 |
| FITC-conjugated anti-mouse CD4 antibody | Biolegend | 100406 |
| APC-conjugated anti-mouse CD25 antibody | Biolegend | 101910 |
| PE anti-mouse FOXP3 antibody | Biolegend | 126404 |
| APC-conjugated anti-mouse IFN-γ antibody | Biolegend | 50581 |
| PE-conjugated anti-mouse IL-4 antibody | Biolegend | 504104 |
| PE/Cyanine7-conjugated anti-mouse IL-17A antibody | Biolegend | 506922 |
| Cell Activation Cocktail | Biolegend | 423304 |
| Foxp3 transcription factor staining buffer kits | eBioscience | 00-5523-00 |
| Recombinant murine IL-2 | PeproTech | 212-12 |
| recombinant murine TGF-β1 | PeproTech | 100-21C |
| recombinant murine IL-12 | PeproTech | 210-12 |
| recombinant Murine IL-1β | PeproTech | 211-11B |
| Anti-murine IFN-γ | PeproTech | 500-P119 |
| Anti-murine IL-4 | PeproTech | 500-P54 |
| AS2863619 | Selleck | S8903 |
| BD Golgi-Plug™ protein transport inhibitor | BD Pharmingen | 555029 |

**Table S2. The sequence of the primers for qRT-PCR**

| **Gene** | **Forward primer** | **Reverse primer** |
| --- | --- | --- |
| TGF-β | ACCATGCCAACTTCTGTCTG | CGGGTTGTGTTGGTTGTAGA |
| CTLA-4 | CCCTGTCTTCTGCAAAGCAAT | ATCATGTAGGTTGCCGCACA |
| GITR | ACGGAAGTGGCAACAACACT | AGCCAAACACAATATCCCCTTGA |
| IRF4 | AGCTCATCACAGCTCATGTGG | TCAGGTAACTCGTAGCCCCT |
| GAPDH | CTGAACGGGAAGCTCACTGG | TGAGGDCCACCACCCTGTTG |

**Table S3. The criteria for clinical score of RA in mice**

| **Score** | **Condition** |
| --- | --- |
| 0 | Normal. |
| 1 | Mild but definite redness of the ankle or wrist, or marked redness restricted to individual fingers. |
| 2 | Moderate redness or swelling of the ankle or wrist. |
| 3 | The entire paw, including the fingers, was severely redness and swelling. |
| 4 | Maximal inflammation of extremities involving multiple joints. |
